# The Constituents of the Stems of *Cissus assamica* and Their Bioactivities

**DOI:** 10.3390/molecules23112799

**Published:** 2018-10-28

**Authors:** Yu-Yi Chan, Chiu-Yuan Wang, Tsong-Long Hwang, Shin-Hun Juang, Hsin-Yi Hung, Ping-Chung Kuo, Po-Jen Chen, Tian-Shung Wu

**Affiliations:** 1 Department of Biotechnology, Southern Taiwan University of Science and Technology, Tainan 71005, Taiwan; yuyichan@stust.edu.tw (Y.-Y.C.); m98h0104@gmail.com (C.-Y.W.); 2 Graduate Institute of Natural Products, School of Traditional Chinese Medicine, College of Medicine, Chang Gung University, Taoyuan 333, Taiwan; htl@mail.cgu.edu.tw; 3 Research Center for Chinese Herbal Medicine, Research Center for Food and Cosmetic Safety, Graduate Institute of Health Industry Technology, College of Human Ecology, Chang Gung University of Science and Technology, Taoyuan 333, Taiwan; 4 Department of Anesthesiology, Chang Gung Memorial Hospital, Taoyuan 333, Taiwan; 5 Department of Pharmacy, Tajen University, Pingtung 90741, Taiwan; paul.juang@gmail.com; 6 School of Pharmacy, College of Medicine, National Cheng Kung University, Tainan 701, Taiwan; z10308005@email.ncku.edu.tw (H.-Y.H.); z10502016@email.ncku.edu.tw (P.-C.K.); 7 Department of Cosmetic Science, Providence University, Taichung 433, Taiwan; litlep@hotmail.com

**Keywords:** Vitaceae, anti-inflammatory, anticancer, cytotoxicity

## Abstract

Fifty-five compounds were isolated from the fresh stems of *Cissus assamica*, including 14 benzenoids, 11 triterpenes, nine steroids, five tocopherols, five chlorophylls, four flavonoids, two benzoquinones, two tannins, and three other compounds. Their structures were constructed by 1D and 2D nuclear magnetic resonance (NMR) and mass spectral data, and were also identified by a comparison of their spectral data with those reported in the literature. Among these isolates, 1,2-bis-(5-γ-tocopheryl) ethane (**51**) was reported for the first time from natural sources. Some purified compounds were examined for their anti-inflammatory and anticancer bioactivities. The results indicated that betulinic acid (**16**) exhibited strong inhibition of superoxide anion generation with IC_50_ value of 0.2 ± 0.1 μM, while betulinic acid (**16**) and pheophytin-a (**47**) inhibited elastase release with IC_50_ value of 2.7 ± 0.3 and 5.3 ± 1.0 μM, respectively. In addition, betulinic acid (**16**) and *epi*-glut-5(6)-en-ol (**18**) exhibited potential cytotoxicity to non-small-cell lung carcinoma (NCI-H226) and colon cancer (HCT-116) cell lines with IC_50_ values in the range of 1.6 to 9.1 μM.

## 1. Introduction

*Cissus assamica* L. belong to the Vitaceae family and is distributed in mainland China, Vietnam, India, Thailand, Indonesia, the southern part of Taiwan, and Lanyu Island [1]. Traditional Chinese medical literature records that the stem of *C. assamica* can activate the circulation to remove blood stasis and treat bruises, fractures, and rheumatoid arthritis [2]. Moreover, several active constituents, such as quinolizidine alkaloids, triterpenes, sterols, flavonoids, stilbenes, and saponins, were isolated and reported from the *Cissus* genus [3,4,5,6,7,8,9,10,11]. Previous biological investigations indicated that the extract of *C. sicyoides* showed moderate cytostatic activity against HEp-2 cells [12] and a significant anti-inflammatory effect [13]. In addition, studies of biological activities also showed hypoglycemic, anti-dyslipidemic, and anti-allergic effects of this genus [14,15,16,17,18,19,20,21,22,23,24,25,26]. However, research related to the species *C. assamica* L. is scarce. Only a few reported the effects of antagonizing the vasoconstriction induced by endothelin-1 [22,23,24,25,26]. Therefore, this study aimed at the purification and identification of anticancer and anti-inflammatory principles from the stem of *C. assamica*.

## 2. Results and Discussion

### 2.1. Isolation and Characterization of Compounds

**Figure 1:**
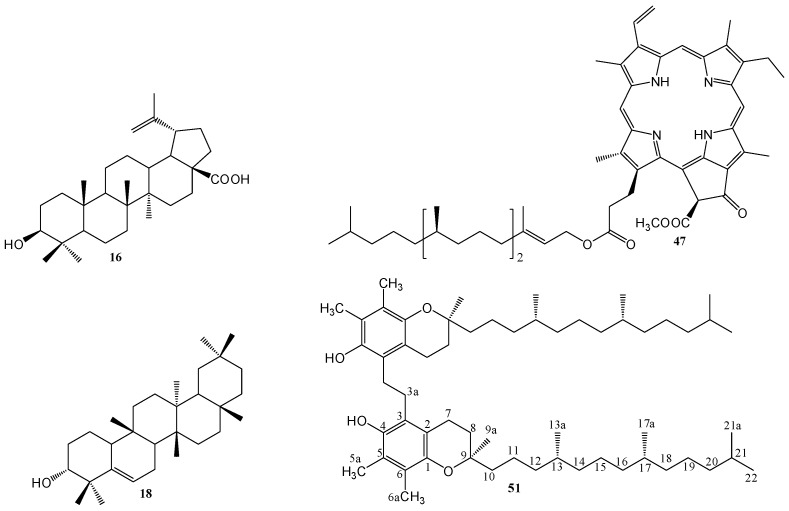
Structures of compounds **16**, **18**, **47** and **51**.

The fresh stems of *C. assamica* L. were extracted with methanol and refluxed for 8 h. The filtrate was concentrated under reduced pressure to yield a dark brown syrup. The crude extract was suspended in water and then partitioned with chloroform and *n*-butanol successively to afford chloroform, *n*-butanol, and water layers, respectively. Purification of the three layers by column chromatography yielded a mixture of β-sitosterol (**1**) and stigmasterol (**2**) [27], β-sitosteryl glucoside (**3**) [28], a mixture of 3β-hydroxyl stigmast-5-en-7-one (**4**) and 3β-hydroxystigmast-5, 22-dien-7-one (**5**) [29], a mixture of β-sitostenone (**6**) [30] and stigmasta-4,22-dien-3-one (**7**) [31], 6β-hydroxy-β-sitostenone (**8**) [32], ergosterol peroxide (**9**) [33], 3,5,7,4′-tetramethoxyflavone (**10**) [34], 3′,4′,3,6,7-pentamethoxyflavone (**11**) [35], 3′,4′,5,6,7-pentamethoxyflavone (**12**) [35], 4′,5,6,7-tetramethoxy-flavone (**13**) [36], a mixture of oleanolic acid (**14**) [30] and ursolic acid (**15**) [28], betulinic acid (**16**) [37], friedelin (**17**) [38], *epi*-glut-5(6)-en-ol (**18**) [39], taraxerol (**19**) [9], *epi*-friedelinol (**20**) [40], glutinone (**21**) [41], lup-28-al-20(29)-en-3-ol (**22**) [42], a mixture of α-amyrin (**23**) and β-amyrin (**24**) [43], bergenin (**25**) [44], *p*-hydroxybenzaldehyde (**26**) [45], vanillin (**27**) [30], methyl gallate (**28**) [46], gallic acid (**29**) [47], 4-methoxybenzoic acid (**30**) [48], vanillic acid (**31**) [30], a mixture of 4-hydroxy-*trans*-cinnamic acid methyl ester (**32**) and 4-hydroxy-*cis*-cinnamic acid methyl ester (**33**) [49], a mixture of octadecyl-*trans*-ferulate (**34**) and octadecyl-*cis*-ferulate (**35**) [50], 1-(4-methoxy-phenyl)undecan-1-one (**36**) [51], 3-hydroxy-4-methoxybenzoic acid (**37**) [52], hexadecyl ferulate (**38**) [45], 2-hydroxybenzoquinone (**39**) [53], 2,6-dimethyoxybenzoquinone (**40**) [49], 3,3′,4-tri-*O*-methyl-ellagic acid (**41**) [54], 3,3′,4,4′-tetra-*O*-methylellagic acid (**42**) [55], methyl pheophorbide-a (**43**) [49], a mixture of methyl-21-hydroxy-(21*S*)-pheophorbide-a (**44**) and methyl-21-hydroxy-(21*R*)-pheophorbide-a (**45**) [49], methyl-21-hydroxyl-(21*S*)-pheophorbide-b (**46**) [49], pheophytin-a (**47**) [49], α-tocopherol (**48**) [56], tocopherol trimer IVa (**49**) [57], tocopherol trimer IVb (**50**) [57], 1,2-bis-(5-γ-tocopheryl)ethane (**51**) [58], α-tocospirol B (**52**) [59], 5,6-dimethoxy-3-methyl-2-cyclohexa-2,5-dien-1,4-dione (**53**) [60], 3-methyl-8-hydroxy-3,4-dihydroisocoumarin (**54**) [61], and methyl linoleate (**55**) [62], respectively. Among them, 1,2-bis-(5-γ-tocopheryl)ethane (**51**) (Figure 1) is reported from natural sources for the first time.

### 2.2. Structural Elucidation of Compound ***51***

1,2-Bis-(5-γ-tocopheryl)ethane (**51**) was isolated as a light yellow syrup. Its UV spectrum had an absorption maximum at 294 nm. The IR spectrum suggested the presence of hydroxyl (3444 cm^−1^) and an aromatic conjugated double bond (1458 and 1377 cm^−1^). The ^13^C-NMR and DEPT spectra exhibited a benzene ring partial structure that has two oxygenated substituents at δ 117.0 (s), 122.1 (s), 123.4 (s), 124.1 (s), 145.5 (s), and 146.3 (s). The ^1^H-NMR spectrum of **51** exhibited signals for two methyl and one methylene groups attached to a benzene ring at δ 2.13, 2.18, and 2.73. Comparing all the ^1^H- and ^13^C-NMR spectral signals carefully, the structure of **51** was similar to that of α-tocopherol (**48**) [56]. It indicated that they are very closely related analogues, differing only in the presence of a methylene group (δ_H_ 2.73, δ_C_ 26.7) in **51**, instead of the methyl group (δ_H_ 2.17, δ_C_ 11.2) found in **48** (Table 1). To establish the structure of **51**, 2D NMR including correlation spectroscopy (COSY), nuclear Overhauser enhancement spectroscopy (NOESY), heteronuclear multiple quantum correlation (HMQC), and heteronuclear multiple bond correlation (HMBC) experiments were conducted. In the HMBC experiment, the correlations observed for H-5a (δ_H_ 2.18)/C-4, C-5, C-6 and H-6a (δ_H_ 2.13)/C-1, C-5, C-6 indicate that two methyl groups are located in the ortho position on the benzene ring. Moreover, the correlation of the methylene proton at δ_H_ 2.73 with C-3 in HMBC spectrum suggests that the location C-3a in the dimerization of alpha-tocopherol forms the dimer **51**. Conclusively, the structure of **51** was assigned as 1,2-bis-(5-γ-tocopheryl)ethane, which had been reported by synthesis [58], but is reported from natural sources for the first time. The NMR spectra are presented Figures S1–S6.

### 2.3. Anti-Inflammatory Activity

Neutrophils are the most abundant white blood cells and participate in the development of the inflammatory reactions in human body; they are important factors in the immune defense against various diseases. Some cytotoxins—for example, the superoxide anion radical, bioactive lipids, granule proteases, and elastase—can be secreted when the different stimuli activate neutrophils. Moreover, they are also major contributors to tissue destruction in chronic inflammatory diseases. It has been proposed that inhibiting neutrophil activation is a method of enhancing inflammatory disorders [63,64,65,66]. Most of the purified compounds in this study were inspected for the inhibition of elastase release and superoxide anion generation by human neutrophils in response to *N*-formyl-l-methionyl-phenylalanine/cytochalasin B (fMLP/CB). Only compound **16** (Figure 1) displayed significant inhibition of superoxide anion generation, with an IC_50_ value of 0.2 ± 0.1 µM (Table 2). In addition, compounds **16** and **47** also exhibited an inhibitory effect on elastase release with an IC_50_ value of 2.7 ± 0.3 and 5.3 ± 1.0 µM, respectively (Table 2). The inhibitory effects of all the tested compounds on superoxide anion generation and elastase release by human neutrophils in response to fMLP/CB are presented in Table S1. The cytotoxicity of compounds **16**, **47**, and LY294002 (a PI3K inhibitor, as a positive control) was examined in human neutrophils using an LDH release assay (Figure S7). All these compounds did not induce LDH release, suggesting that the inhibitory effects did not result from cytotoxicity in human neutrophils.

### 2.4. Cytotoxicity

In order to evaluate the growth inhibitory activity of the purified compounds against cancer cells, this study selected three different cell lines from malignant tumors including human nasopharyngeal carcinoma (NPC-TW01), non-small-cell lung carcinoma (NCI-H226), and colon cancer cell lines (HCT116). The results showed that betulinic acid (**16**) and *epi*-glut-5(6)-en-ol (**18**) (Figure 1) exhibited significant cytotoxicity with IC_50_ values ranged from 1.6 to 9.1 μM (Table 3). Moreover, betulinic acid (**16**) exhibited powerful inhibitory activity against NCI-H226 and HCT116 with IC_50_ values of 2.0 and 1.6 μM, respectively. Our study suggested the stem extracts of *C. assamica* and the purified compounds are potential candidates for the development of anti-cancer drugs. The preliminary growth inhibitory activity of all the tested compounds is presented in Table S2.

## 3. Materials and Methods

### 3.1. General Information

UV spectra were obtained with a Hitachi UV-3210 and UV-3010 spectrophotometer (Hitachi, Tokyo, Japan), and IR spectra were measured with a Shimadzu FTIR Prestige-21 spectrometer (Shimadzu, Kyoto, Japan). Optical rotations were measured with a HORIBA SEPA-300 digital polarimeter in a 0.5 dm cell (Horiba, Kyoto, Japan). The ESIMS and HRESIMS were taken on a Bruker Daltonics APEX II 30e spectrometer (Bruker, Billerica, MA, USA). ^1^H- and ^13^C-NMR spectra were measured using Bruker Avance-300, AMX-400, and AV-500 spectrometers (Bruker, Billerica, MA, USA) with TMS as the internal reference, and chemical shifts are expressed in δ (ppm). Silica gel (70–230 and 230–400 mesh; Merck, Darmstadt, Germany) and Spherical C18 100 Å reversed phase silica gel (RP-18; particle size 20–40 μm; Silicycle, Quebec City, QC, Canada) were used for column chromatography (CC), and silica gel 60 F_254_ and RP-18 F_254S_ thin-layer chromatography (TLC) plates (Merck, Darmstadt, Germany) were used for preparative TLC, respectively.

### 3.2. Materials

The fresh stems of *C. assamica* L. were collected from Taitung Hsien, Taiwan, in October 2009 and verified by Prof. Chang-Sheng Kuoh (Department of Biology, National Cheng Kung University, Tainan, Taiwan). A voucher specimen (TSWu 20091016) has been deposited in the Herbarium of School of Pharmacy, National Cheng Kung University, Tainan, Taiwan. 

### 3.3. Extraction and Isolation

The fresh stems of *C. assamica* L. (15 kg) were extracted with methanol (15 × 20 L) and refluxed for 8 h. The filtrate was evaporated under reduced pressure to yield a dark brown syrup (418 g). The residue was suspended in water and then partitioned with chloroform (5 × 2 L) and *n*-butanol (5 × 2 L) successively to afford chloroform (63 g), *n*-butanol (145 g) and water (210 g) soluble fractions respectively.

The chloroform soluble extracts were fractionated via silica gel column chromatography eluting with *n*-hexane/acetone (9:1) to afford seven fractions, on the basis of TLC monitoring. Fraction 1 was subjected to silica gel column chromatography eluted with *n*-hexane/acetone (79:1) to yield a mixture of β-sitosterol (**1**) and stigmasterol (**2**) (3.1 g), 3,5,7,4′-tetramethoxyflavone (**10**, 2.9 mg), 3′,4′,3,6,7-pentamethoxyflavone (**11**, 12.2 mg), 3′,4′,5,6,7-pentamethoxyflavone (**12**, 6.1 mg), 4′,5,6,7-tetra-methoxyflavone (**13**, 3.1 mg), friedelin (**17**, 1.8 g), taraxerol (**19**, 3.9 mg), glutinone (**21**, 3.2 mg) and methyl linoleate (**55**, 20.7 mg).

Purification of fraction 2 by column chromatography with silica gel was eluted by a gradient of benzene/ethyl acetate (79:1) to afford *epi*-glut-5(6)-en-ol (**18**, 23.2 mg), α-tocopherol (**48**, 200 mg), tocopherol trimer IVa (**49**, 24.3 mg), tocopherol trimer IVb (**50**, 28.1 mg), 1,2-bis-(5-γ-tocopheryl)- ethane (**51**, 15.4 mg), α-tocospirol B (**52**, 6.1 mg) and 5,6-dimethoxy-3-methyl-2-cyclohexa-2,5-dien-1,4-dione (**53**, 52.2 mg).

Separation of fraction 3 by column chromatography with silica gel eluted by *n*-hexane/ethyl acetate (9:1) yielded a mixture of 3β-hydroxystigmast-5-en-7-one (**4**) and 3β-hydroxystigmast- 5,22-dien-7-one (**5**, 4.5 mg), a mixture of β-sitostenone (**6**) and stigmasta-4,22-dien-3-one (**7**, 13.7 mg), 6β-hydroxy-β-sitostenone (**8**, 16.9 mg), ergosterol peroxide (**9**, 35 mg), *epi*-friedelinol (**20**, 200 mg), lup-28-al-20(29)-en-3-ol (**22**, 3.2 mg), a mixture of α-amyrin (**23**) and β-amyrin (**24**, 60 mg), a mixture of 4-hydroxy-*trans*-cinnamic acid methyl ester (**32**) and 4-hydroxy-*cis*-cinnamic acid methyl ester (**33**, 190 mg), a mixture of octadecyl-*trans*-ferulate (**34**) and octadecyl-*cis*-ferulate (**35**, 45.3 mg), hexadecyl ferulate (**38**, 20.7 mg) and 3-methyl-8-hydroxy-3,4-dihydroisocoumarin (**55**, 3.5 mg).

Fraction 4 was chromatographed over silica gel eluted with a benzene/acetone gradient (49:1) to give a mixture of oleanolic acid (**14**) and ursolic acid (**15**, 49.6 mg), betulinic acid (**16**, 63.6 mg), 4-methoxybenzoic acid (**30**, 4.7 mg), 1-(4-methoxyphenyl)undecan-1-one (**36**, 2.3 mg) and pheophytin-a (**47**, 11.2 mg).

Purification of fraction 5 by column chromatography with silica gel was eluted by a gradient of chloroform/acetone (49:1) to afford 2-hydroxybenzoquinone (**39**, 3.5 mg), methyl pheophorbide-a (**43**, 3.1 mg), a mixture of methyl-21-hydroxy-(21*S*)-pheophorbide-a (**44**) and methyl-21-hydroxy-(21*R*)-pheophorbide-a (**45**, 3.5 mg).

Separation of fraction 6 by column chromatography with a silica gel eluted by chloroform/acetone (49:1) yielded *p*-hydroxybenzaldehyde (**26**, 4.0 mg), 3,3′,4-tri-*O*-methylellagic acid (**41**, 37.7 mg), 3,3′,4,4′-tetra-*O*-methylellagic acid (**42**, 1.5 mg) and methyl-21-hydroxy-(21*S*)-pheophorbide-b (**46**, 3.6 mg).

Fraction 7 was subjected to silica gel column chromatography eluted with chloroform/methanol (49:1) to yield β-sitosteryl glucoside (**3**, 700 mg), vanillin (**27**, 7.8 mg), vanillic acid (**31**, 1.7 mg), 3-hydroxy-4-methoxybenzoic acid (**37**, 3.2 mg), 2, 6-dimethyoxybenzoquinone (**40**) (3.5 mg).

The *n*-butanol layer was subjected directly to Diaion HP-20 column chromatography, eluted with water containing increasing proportions of methanol, to give six fractions. Fraction 1 was chromatographed over Sephadex LH-20 eluted with gradient of water/methanol to give gallic acid (**29**, 600 mg). Fraction 2 was chromatographed on Sephadex LH-20 eluted with gradient of water/methanol to afford bergenin (**25**, 6.2 g). Fraction 4 was chromatographed on Sephadex LH-20 with water/methanol to give methyl gallate (**28**, 32.1 mg).

1,2-Bis-(5-γ-tocopheryl)ethane (**51**): light yellow syrup; UV λ_max_ (MeOH) nm (log ε) 294; IR (KBr) ν_max_ cm^−1^ 3444, 2920, 2850, 1458, 1377, 1257, 1087; ^1^H- and ^13^C-NMR data, see Table 1.

### 3.4. Anti-Inflammatory Bioactivity Examination

#### 3.4.1. Preparation of Human Neutrophils

Human neutrophils study was approved by Chang Gung Memorial Hospital Institutional Review Board, Taoyuan, Taiwan. It was conducted according to the Declaration of Helsinki. Blood was obtained from healthy donors (20–32 years old) who provided written informed consent before blood was drawn. Briefly, neutrophils were isolated by dextran sedimentation, Ficoll‒Hypaque gradient centrifugation, and hypotonic lysis of the erythrocytes [67].

#### 3.4.2. Measurement of Superoxide Anion Generation and Elastase Release

The superoxide anion generation and elastase release were measured using the reduction of ferricytochrome *c* and elastase substrate, methoxysuccinyl-Ala-Ala-Pro-Val-*p*-nitroanilide, respectively, as described previously [68,69,70]. Human neutrophils were suspended in HBSS containing ferricytochrome *c* (0.6 mg/mL) or elastase substrate (100 μM) at 37 °C and treated with DMSO or tested compounds for 5 min. The cells were then activated using fMLF (0.1 μM)/cytochalasin B (CB, 1 μg/mL for superoxide generation and 0.5 μg/mL for elastase release) and the change of absorbance was continually measured at 550 nm and 405 nm by a spectrophotometer (U-3010, Hitachi) to determine the superoxide anion generation and elastase release, respectively.

#### 3.4.3. Detection of Cytotoxicity

Human neutrophils were treated with DMSO or tested compounds and incubated at 37 °C for 15 min. The supernatant was assayed to detect the released LDH using CytoTox 96 non-radioactive cytotoxicity assay (Promega, Madison, WI, USA). The results are presented in Figure S7.

### 3.5. Determination of Anticancer Bioactivity

#### 3.5.1. Cell Lines

Human cancer cell lines, non-small cell lung carcinoma (NCI-H226) and colon cancer cell line (HCT116) were obtained from the American Type Culture Collection (Rockville, MD, USA). A nasopharyngeal carcinoma (NPC-TW01) cell line was purchased from Food Industry Research and Development Institute (Hsinchu, Taiwan). Tumor cells were maintained in proper medium supplemented with 10% fetal bovine serum (FBS) at 37 °C in a humidified atmosphere of 5% CO_2_.

#### 3.5.2. Growth Inhibition Assay

The evaluation of cell growth and survival was carried out according to Hansen et al. [71] with some modifications.

## 4. Conclusions

In summary, 55 compounds were characterized from the fresh stems of *C. assamica*, including 14 benzenoids, 11 triterpenes, nine steroids, five tocopherols, five chlorophylls, four flavonoids, two benzoquinones, two tannins, and three other compounds. Among these isolates, 1,2-bis-(5-γ-tocopheryl)ethane was reported for the first time from natural sources. Furthermore, the inhibitory activity on superoxide anion generation and elastase release and the cytotoxicity on three cancer cells were analyzed. The present study suggests that the stems of *C. assamica* and several compounds of its isolation could be further developed as candidates for the treatment or prevention of cancer and various inflammatory diseases. Thus, the detailed mechanism of action of these compounds appears worthy of follow-up investigation.

## Figures and Tables

**Table 1:** ^1^H- and ^13^C-NMR spectra data of **48** and **51** (CDCl_3_, 400 MHz).

Position	48		51	
	δ_H_ (mult., *J* in Hz)	δ_C_	δ_H_ (mult., *J* in Hz)	δ_C_
1		145.5		146.3
2		117.2		117.0
3		118.5		123.4
4		144.5		145.5
5		121.1		122.1
6		122.6		124.1
7	2.64 (t, 4.5)	21.0	2.74 (m)	21.7
8	1.79 (m)	31.5	1.83 (m)	32.3
9		74.5		75.3
10	1.56 (m)	39.8	1.56 (m)	40.7
11	1.54 (m)	22.6	1.54 (m)	21.3
12	1.26~1.25 (m)	37.4	1.28~1.25 (m)	38.2
13	1.31 (d, 7.4)	32.7	1.32 (d, 7.2)	33.4
14	1.26~1.25 (m)	37.2	1.28~1.25 (m)	37.9
15	1.26~1.25 (m)	24.8	1.28~1.25 (m)	25,5
16	1.26~1.25 (m)	37.5	1.28~1.25 (m)	38.2
17	1.31 (d, 7.4)	32.8	1.32 (d, 7.2)	33.5
18	1.16~1.13 (m)	37.4	1.16~1.13 (m)	38.2
19	1.16~1.13 (m)	25.1	1.16~1.13 (m)	25,2
20	1.16~1.13 (m)	39.4	1.16~1.13 (m)	40.1
21	1.52 (m)	27.9	1.52 (m)	28.7
22	0.91 (d, 6.4)	23.7	0.86 (d, 7.2)	23.4
3a	2.17 (s)	11.2	2.73 (s)	26.7
5a	2.22 (s)	12.8	2.18 (s)	12.8
6a	2.19 (s)	11.8	2.13 (s)	12.6
9a	1.28 (s)	24.4	1.24 (s)	24.5
13a	0.90 (d, 7.4)	20.3	0.85 (d, 7.2)	20.3
17a	0.89 (d, 7.4)	20.7	0.83 (d, 7.2)	20.5
21a	0.93 (d, 6.5)	22.7	0.87 (d, 7.2)	23.3
OH	4.25 (s)		5.41 (s)	

**Table 2:** Inhibitory effects of isolated compounds on superoxide anion generation and elastase release by human neutrophils in response to fMLP/CB.

Compound	Superoxide Anion Generation	Elastase Release
	IC_50_ (μM) ^a^	IC_50_ (μM)
**16**	0.2 ± 0.1 ***	2.7 ± 0.3 ***
**47**	>10	5.3 ± 1.0 ***
**LY294002** ^b^	0.4 ± 0.1 ***	1.5 ± 0.3 ***

Results are presented as mean ± S.D. (*n* = 3~4). *** *p* < 0.001 compared with the control (DMSO). ^a^ Concentration necessary for 50% inhibition (IC_50_). ^b^ A phosphatidylinositol-3-kinase inhibitor was used as a positive control.

**Table 3:** Cytotoxicity of compounds **16**, **18**, **20**, **21**, **41** and **52**.

Compounds	Cell Lines	
	NCI-H226	HCT-116
	IC_50_ (μM)	IC_50_ (μM)
**16**	2.0	1.6
**18**	9.1	6.0
**20**	15.8	16.7
**21**	38.0	24.0
**41**	31.6	30.3
**52**	>50	39.4

## Supplementary Materials

The following are available online. Tables S1 and S2: Anti-inflammatory and cytotoxic effects of all the tested compounds from *C. assamica*; Figures S1–S6: NMR spectra of compound **51**; Figure S7: Cytotoxicity of compounds **16**, **47** and LY294002.
