# Supplementary material for: The Constituents of the Stems of Cissus assamica and Their Bioactivities
Source: Molecules. 2018 Oct 28;23(11):2799. doi: 10.3390/molecules23112799 (PMC6278371; doi:10.3390/molecules23112799)
Supplement: Supplementary file 1 [file molecules-23-02799-s001.pdf]

Supporting Information

The Constituents of the Stems of *Cissus assamica* and Their Bioactivities

Yu-Yi Chan [1], Chiu-Yuan Wang [1], Tsong-Long Hwang [2], Shin-Hun Juang [3], Hsin-Yi Hung [4], Ping-Chung Kuo [4], Po-Jen Chen [5], Tian-Shung Wu [3,4,*]

[1] Department of Biotechnology, Southern Taiwan University of Science and Technology, Tainan 71005, Taiwan

[2] Graduate Institute of Natural Products, College of Medicine, Chang Gung University; Research Center for Industry of Human Ecology, Research Center for Chinese Herbal Medicine, and Graduate Institute of Health Industry Technology, Chang Gung University of Science and Technology; Department of Anesthesiology, Chang Gung Memorial Hospital, Taoyuan 333, Taiwan

[3] Department of Pharmacy, Tajen University, Pingtung 90741, Taiwan

[4] School of Pharmacy, College of Medicine, National Cheng Kung University, Tainan 701, Taiwan

[5] Department of Cosmetic Science, Providence University, Taichung 433, Taiwan

* Correspondence: tswu@mail.ncku.edu.tw; Tel.:+886-6-2757575 (ext. 65333).

Table S1. Inhibitory effects of isolated compounds on superoxide anion generation and elastase release by human neutrophils in response to fMLP/CB.

compound	superoxide anion generation	elastase release
	IC_{50} (μM)[a]	IC_{50} (μM)
**3**	>10	>10
**8**	>10	>10
**9**	>10	>10
**16**	0.2 ± 0.1 ***	2.7 ± 0.3 ***
**17**	>10	>10
**18**	>10	>10
**20**	>10	NT[c]
**25**	>10	>10
**28**	>10	>10
**29**	>10	>10
**41**	>10	>10
**47**	>10	5.3 ± 1.0 ***
**48**	>10	>10
**51**	>10	>10
**LY294002**[b]	0.4 ± 0.1 ***	1.5 ± 0.3 ***

Results are presented as mean ± S.D. (n = 3~4). ****p* < 0.001 compared with the control (DMSO). [a] Concentration necessary for 50 % inhibition (IC_{50}). [b] A phosphatidylinositol-3-kinase inhibitor was used as a positive control. [c] The compound reacted with the substrate, and caused the absorbance greater than 0.2. Therefore, it was not measured the data.

Table S2. The IC_{50} of cancer cell lines treated with CAS Drugs (Tested at 50, 10 μM)

Compounds \ Cell Lines	NCI-H226	HCT-116	NPC-TW01
	IC_{50} (μM)	IC_{50} (μM)	IC_{50} (μM)
**3**	>50	>50	>50
**8**	>50	>50	<50
**9**	>50	>50	<50
**11**	>50	>50	>50
**16**	<10	<50	>50
**17**	>50	>50	>50
**18**	<10	<50	>50
**20**	<10	<50	>50
**21**	>50	<50	<50
**22**	>50	>50	>50
**25**	>50	>50	>50
**28**	>50	>50	>50
**29**	>50	<50	>50
**30**	>50	>50	>50
**37**	>50	>50	>50
**38**	>50	>50	>50
**39**	>50	>50	>50
**40**	>50	>50	>50
**41**	>50	<50	<50
**47**	>50	>50	>50
**48**	>50	>50	>50
**49**	>50	>50	>50
**50**	>50	>50	>50
**51**	>50	>50	>50
**52**	>50	<50	<50
**53**	>50	>50	>50

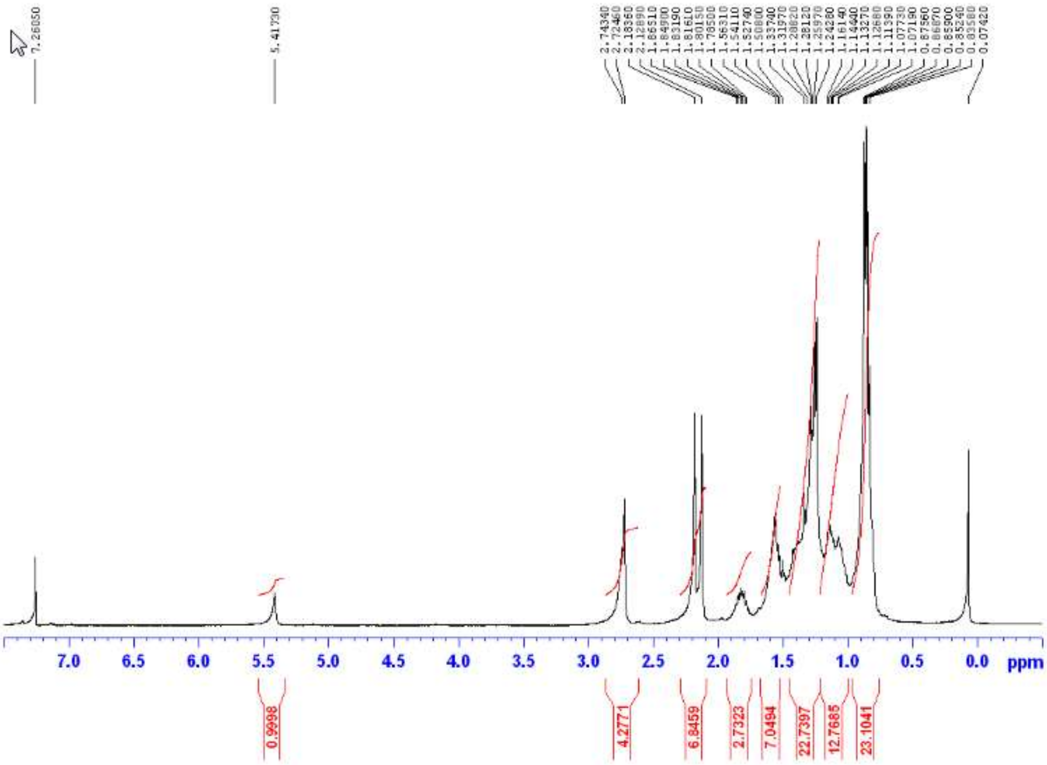

Figure S1. The ^{1}H spectrum of 1, 2-bis-(5-γ-tocopheryl)ethane (**51**)

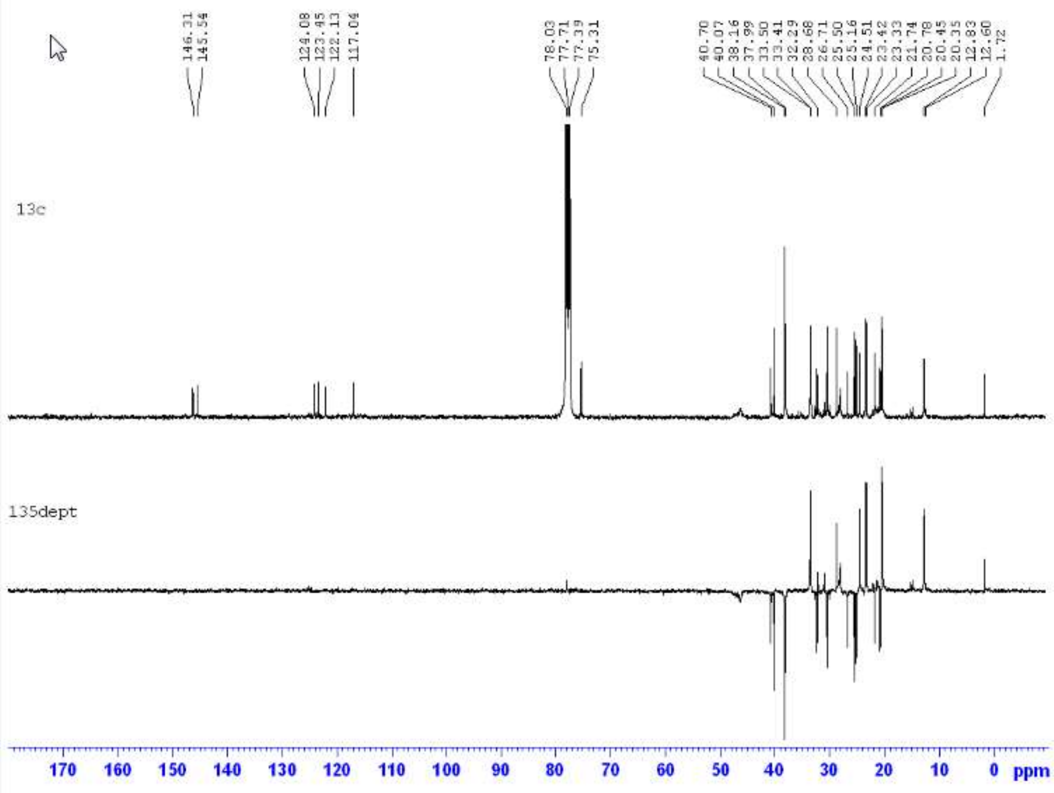

Figure S2. The ^{13}C and DEPT spectra of 1, 2-bis-(5-γ-tocopheryl)ethane (**51**)

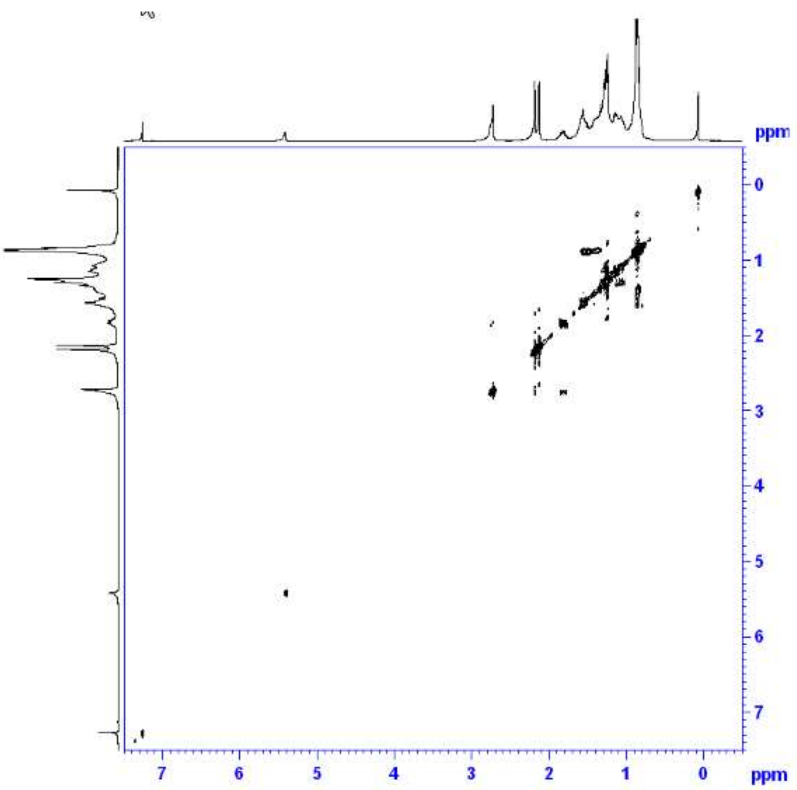

Figure S3. The COSY spectrum of 1, 2-bis-(5-γ-tocopheryl)ethane (**51**)

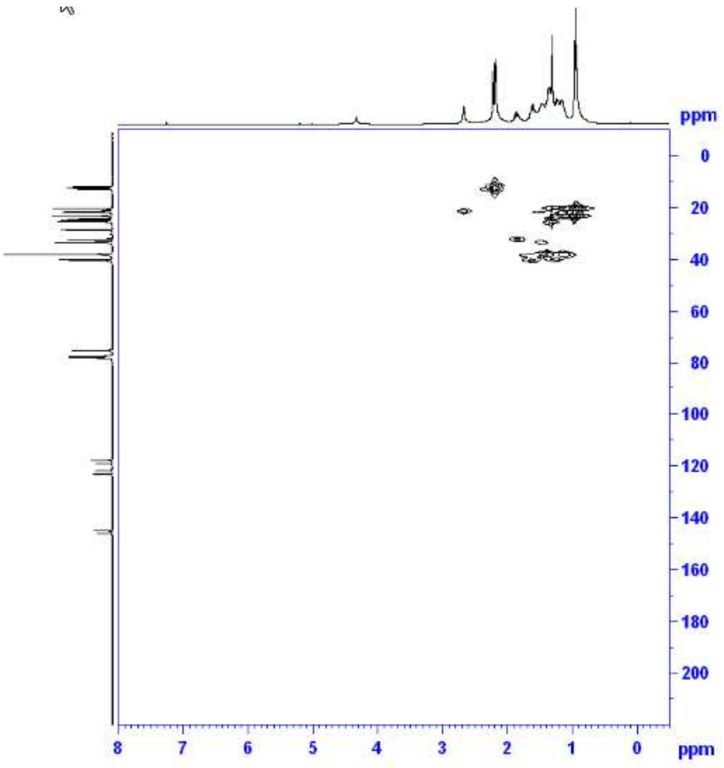

Figure S4. The HMQC spectrum of 1, 2-bis-(5-γ-tocopheryl)ethane (**51**)

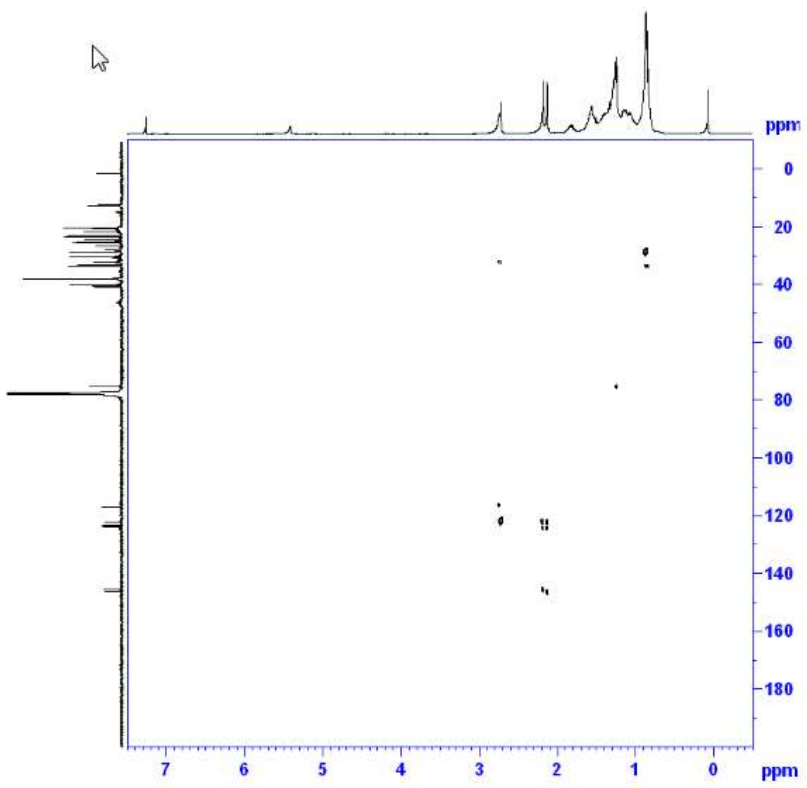

Figure S5. The HMBC spectrum of 1, 2-bis-(5-γ-tocopheryl)ethane (**51**)

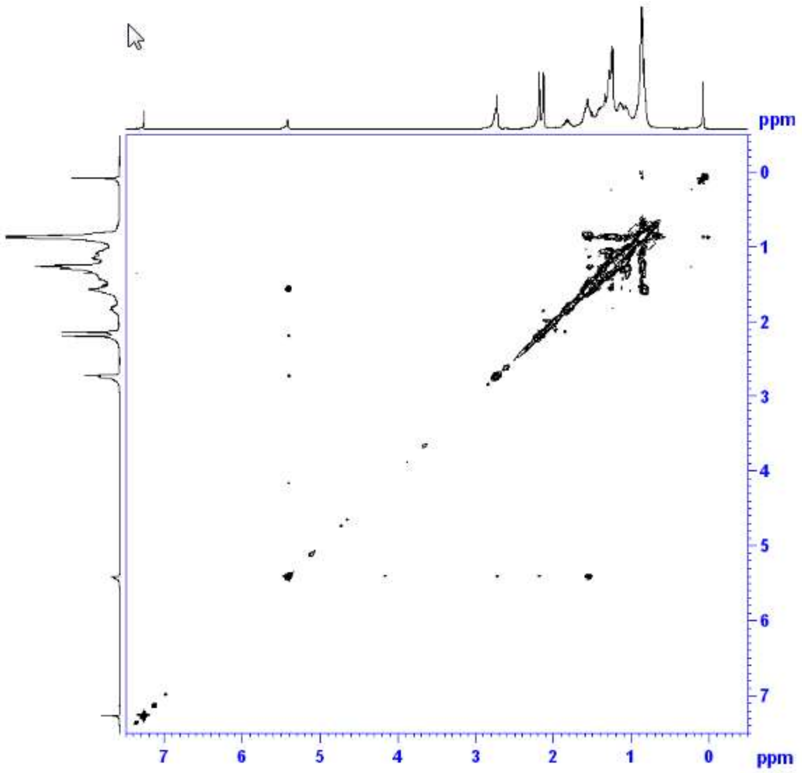

Figure S6. The NOESY spectrum of 1, 2-bis-(5-γ-tocopheryl)ethane (**51**)

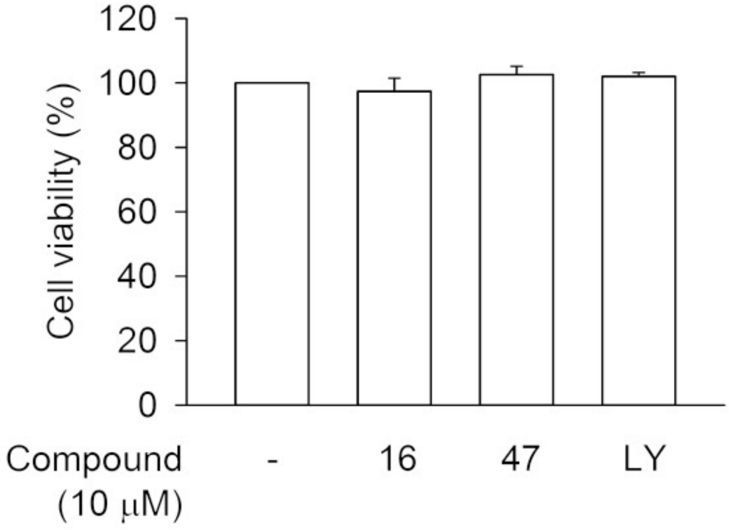

Figure S7. Compounds **16**, **47**, and LY294002 do not alter cell viability of human neutrophils. Human neutrophils were incubated with DMSO, compounds **16**, **47**, or LY294002 (10 μM) for 15 min. Cytotoxicity was evaluated by LDH release compared with the DMSO group (as 100%). All data are expressed as mean values ± SEM (n = 3).
